# Acute Myopericarditis in the Setting of Crohn’s Colitis: Challenging Management Decisions

**DOI:** 10.7759/cureus.22794

**Published:** 2022-03-03

**Authors:** Jordan Daloya, Aqsa Ashraf, Alan Kaell, Rohan Perera, Giridhar Korlipara

**Affiliations:** 1 Internal Medicine, Zucker School of Medicine/Northwell Health, Mather Hospital, Port Jefferson, USA; 2 Rheumatology, Zucker School of Medicine/Northwell Health, Mather Hospital, Port Jefferson, USA; 3 Cardiology, Zucker School of Medicine/Northwell Health, Mather Hospital, Port Jefferson, USA; 4 Medicine/Cardiology, Zucker School of Medicine/Northwell Health, Mather Hospital, Port Jefferson, USA

**Keywords:** mesalamine, tnf alpha inhibitor, inflammatory bowel disease, extra-intestinal manifestations, crohns, acute myopericarditis

## Abstract

Myopericarditis is a rare extraintestinal manifestation of Crohn’s disease (CD). Myopericarditis has also been attributed to treatment with mesalamine and heart failure to tumor necrosis factor inhibitor (TNFi) use. When a patient with CD, controlled on these medications, presents with myopericarditis and/or heart failure, it can confound both the differential diagnosis and management of such patients. Our case is acute myopericarditis in a 34-year-old male, with a history of CD controlled with mesalamine and infliximab, who had been off TNFi therapy for over six months due to loss of insurance coverage and had been intermittently using leftover mesalamine. He presented to the ED complaining of a one-day history of abdominal pain with bloody diarrheal stools, chest discomfort, and fever. A colonoscopy performed two days back had demonstrated active colonic CD. Findings included ECG evidence of pericarditis, elevated cardiac biomarkers, and reduced left ventricular function on ventriculography consistent with myopericarditis. We present the differential, diagnostic and management challenges encountered in this situation, review the pertinent literature, and discuss decision making in what appears to be myopericarditis attributed to an extraintestinal manifestation of active GI Crohn’s.

## Introduction

Systemic involvement in Crohn’s disease (CD) affecting the skin, musculoskeletal, and hepatobiliary systems is well known. Cases of myopericarditis are rarely seen and have been attributed to extraintestinal CD. [1] However, some therapies commonly prescribed for CD such as mesalamine and tumor necrosis factor inhibitor (TNFi) have reported, respectively, adverse effects (AEs) of myocarditis/pericarditis and heart failure [2,3]. This possibility makes the differential diagnosis challenging for acute and chronic CD patients on such medical management who may present with these conditions. Is it an AE of therapy or an extraintestinal manifestation of CD? We describe a case of a male with a CD exacerbation, who developed acute myopericarditis with management confounded concerns for heart failure, myopericarditis, and possible infection.

## Case presentation

A 34-year-old male with a history of CD presented to the ED with a one-day history of high-grade fever with chills, crampy abdominal pain, and bloody diarrheal stools with mucous. Additionally, he complained of persistent, non-radiating, mild substernal chest pressure. He denied any prior history of similar chest pain in the past, and he had a family history of a heart attack in his uncle at the age of 40. He described his symptoms beginning two days after colonoscopy, with biopsies for suspected disease activity. He had been off his chronic medication (infliximab) for 10 months due to a lack of health insurance coverage. He took mesalamine intermittently until presentation. Vital signs were significant for a heart rate of 120 beats per minute, a temperature of 103 Fahrenheit, and a blood pressure of 98/82 mmHg. Physical exam was notable for normal heart sounds without gallop, no jugular vein distention, and Kussmaul's sign or pericardial rubs. His lungs were clear to auscultation. The abdomen was minimally tender throughout though most prominent in the left lower quadrant without rebound and normal bowel sounds. 

**Figure 1:**
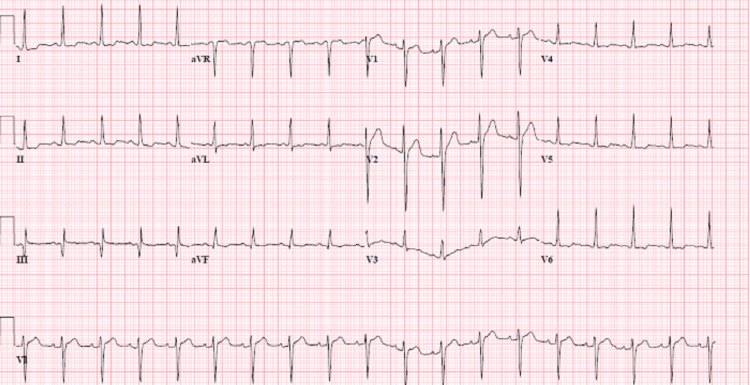
First ECG obtained on presentation showing sinus tachycardia with non-specific ST-segment changes in lateral leads.

IV hydration was initiated, and the patient was kept nil per os. Admission laboratory tests, including complete blood count, complete metabolic panel, and serum troponin, were within normal range. Initial ECG (Figure 1) showed sinus tachycardia with non-specific ST changes in the inferior leads, and chest X-ray was unremarkable. CT of the abdomen and pelvis with IV contrast showed mild colonic wall thickening extending from the ascending to the sigmoid colon. He was started on empiric antibiotic therapy IV hydration and was admitted for colitis with differentials being CD flare versus infection. After a team discussion between gastroenterologists, cardiologists, and infectious disease specialists, a decision was made to assess the response to antibiotic therapy alone.

**Figure 2:**
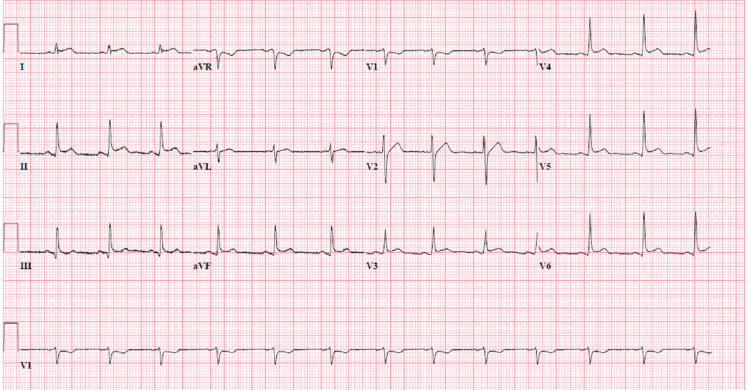
Normal sinus rhythm with ST-segment elevations evident in lateral and inferior leads. Non-specific ST segment abnormality in precordial leads.

**Figure 3:**
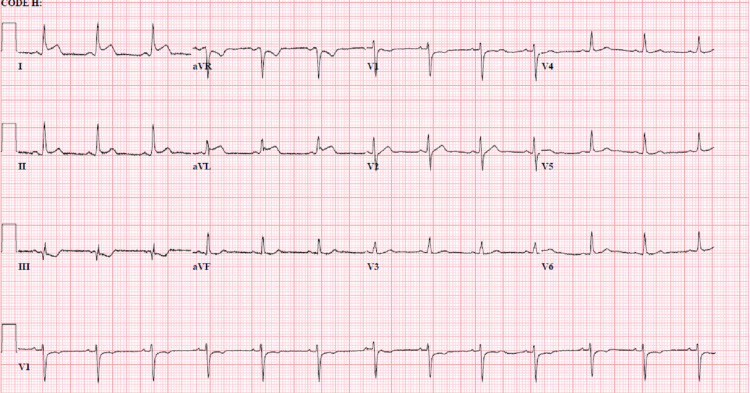
Sinus rhythm and acute ST-segment elevations now present in lateral leads.

The next day, he complained of worsening substernal chest discomfort unaffected by changing positions from supine to leaning forward. He had some nausea without emesis, but no paroxysmal nocturnal dyspnea, dyspnea on exertion, or shortness of breath at rest. IV morphine was only minimally effective in symptom relief. Repeat ECG (Figure 2) showed sinus rhythm with acute ST-segment elevations in leads 1 and augmented Vector Left (aVL), and high sensitivity cardiac troponin (hscTn) elevation to 685 ng/L (nl <= 20 ng/L). He received high-dose aspirin, a loading dose of ticagrelor, and was started on a heparin drip. Urgent cardiac catheterization showed normal coronaries with evidence of reduced left ventricular ejection fraction (LVEF) of 35% and severe apical hypokinesis. Post catheterization, he continued to have chest discomfort. Serial ECGs were obtained that showed diffuse ST-segment elevations suggestive of acute pericarditis (Figure 3). The following day, a transthoracic echocardiogram (TTE) showed a normal LVEF of 63% without evidence of significant pericardial effusion. HscTn was noted to peak at 4066 ng/L. With ECG evidence of acute pericarditis and a large hscTn leak, the diagnosis of acute myopericarditis was made, and the patient was started on indomethacin and colchicine, resulting in symptomatic relief within one day. Blood and stool cultures, urine analysis, viral serology including Epstein-Barr virus and cytomegalovirus, Clostridium difficile, and stool ova and parasite, were all negative, implicating the myopericarditis as an extra-GI manifestation of his active CD flares. Following clinical improvement, negative purified protein derivative (PPD) study, and confirmation of TTE with normal LVEF, adalimumab therapy was initiated, and social services arranged for financial support for his medication.

## Discussion

Inflammatory bowel disease (IBD), with its two major subgroups of CD and ulcerative colitis (UC), is a chronic idiopathic disorder of inflammation of the GI tract. In 2017, there were 6·8 million cases of IBD reported globally, with the highest age-standardized prevalence rate occurring in North America [4]. IBD is most commonly diagnosed between the ages of 15 and 30 years. However, it has been described as having a bimodal age distribution with a possible second peak at the age of 50-80 [5]. Extraintestinal manifestations (EIMs) can be seen in 25-40% of IBD patients with primary manifestations involving the skin, joints, liver, and eyes [6]. Cardiac involvement like pericarditis and myocarditis is rarely reported as IBD manifestations [1]. However, as compared to the general population, the incidence of myocarditis has been reported to be higher among patients with IBD [7]. Additionally, rare cases of myopericarditis have been related to mesalamine, first-line therapy for mild-to-moderate CD. Sulfasalazine was the first drug marketed for the treatment of mild-to-moderate IBD, with its active component being 5-aminosalicylic acid (5-ASA), mesalamine, bound to a sulfapyridine molecule. This drug was shown to have a rare but potentially fatal AE of cardiac toxicity, involving the pericardium, myocardium, or both. Thoughts at the time were that the sulfapyridine molecule was the cause, but cases of cardiotoxicity followed with formulations of 5-ASA alone were then considered the causes. Though unproven, possible mechanisms that can explain the cardiotoxic effects include a direct toxic effect from the drug, an allergic reaction mediated by IgE, hypersensitivity reaction (cell-mediated), or a humoral response [8]. 

Clinical presentations of acute myocarditis most often include acute onset chest pain, palpitations, progressive dyspnea at rest or exertion, and cardiogenic shock signs. The current gold standard for diagnosis of myocarditis is endomyocardial biopsy which is rarely performed. Therefore, criteria for noninvasive diagnostic have been established for clinically suspected myocarditis based on clinical presentations and diagnostic tools including ECG, TTE, cardiac biomarkers, and cardiovascular magnetic imaging. ECG abnormalities may include evidence of new arrhythmias, acute ST changes, or conduction abnormalities. Echocardiographic imaging may suggest evidence of new-onset heart failure. One or more clinical features and more diagnostic features suggest clinically suspected myocarditis [9]. 

Management of myopericarditis varies based on the etiology and degree of myocardial involvement [10]. Empiric first-line therapy for the initial episode of myopericarditis is nonsteroidal anti-inflammatory drug (NSAID) therapy. If NSAID therapy is contraindicated or poorly tolerated, prednisone therapy would be an appropriate alternative. Colchicine, used as adjuvant therapy in the setting of acute or recurrent pericarditis, is often utilized in cases of myopericarditis though data is limited to support its efficacy [11]. Until heart failure concerns resolve, TNFi should be avoided as it may contribute to further decompensation [2]. Therefore, outpatient monitoring and continued surveillance of cardiac function should continue following initial presentation prior to initiation of TNFi therapy.

## Conclusions

Our patient's clinical presentation warrants a differential diagnostic consideration of multiple entities, including ischemic, metabolic, infectious, and pharmacological etiologies. Ischemic, infectious, and metabolic conditions were ruled out. Stress cardiomyopathy was considered very unlikely given the patient's gender, age, and the absence of an antecedent stressful event. We also did not attribute his myopericarditis to the sporadic and sparing use of mesalamine, although rare case reports in the literature suggest it cannot be excluded. However, in the setting of his acute CD flare and otherwise negative workup, an associated autoimmune process as an extraintestinal manifestation of CD was the most likely etiology.

Myopericarditis is a rare but potentially catastrophic result of acute CD flare and its therapies associated with management. Timely assessment and recognition of this rare extraintestinal manifestation of IBD is essential as signs and symptoms may be subtle and easily overlooked. A well-orchestrated multidisciplinary outpatient surveillance team involving primary care, cardiology, and gastroenterology is essential to reduce the risk of progression and recurrence.
